# Parent-child attachment in children born preterm and at term: A multigroup analysis

**DOI:** 10.1371/journal.pone.0202972

**Published:** 2018-08-30

**Authors:** Nina Ruiz, Bernhard Piskernik, Andrea Witting, Renate Fuiko, Lieselotte Ahnert

**Affiliations:** 1 Department of Applied Psychology: Health, Development, Enhancement and Intervention, Faculty of Psychology, University of Vienna, Vienna, Austria; 2 University Children´s Hospital Vienna, Vienna, Austria; University of Liverpool, UNITED KINGDOM

## Abstract

**Objective:**

While ample research exists about mother-child attachment, so far little focus has been on specifics of father-child attachment. Even less research is available on the nature of the father-child relationship for children born preterm. The objective of this study was to determine whether children born preterm (23 to 37 weeks gestation) differ in their attachment to their fathers and mothers from their term peers (> 37 weeks gestation), and whether specific child characteristics, such as gender, twin status, and developmental status, have an influence on the parent-child relationship.

**Methods:**

The sample consisted of 290 children (n = 140 girls, 48.28%), 190 born before term (including 45 twin pairs) between 12 and 36 months of age (*M* = 19.5, *SD* = 5.7) and 100 term children of the same age (*M* = 18.8, *SD* = 6.1) with their 245 fathers and mothers. Attachment of the children with their mothers and fathers was assessed using the Attachment Q-sort during two home visits. Children’s developmental status was measured with the Bayley Scales of Infant and Toddler Development.

**Results:**

Within a multigroup analysis of parents with children born preterm and at term more secure attachment was found for both parents in the term sample than in the preterm group. Correlates of attachment specific to fathers of children born preterm accumulated to an explained variance of *R*^2^ = .82. For those fathers, less education as well as lower development scores and male gender of the child were associated with lower attachment scores. In the three other parent-child constellations the explained variance did not exceed 20%. Child development proved to be a significant predictor for father-child attachment regardless of the child’s birth status. Male gender was associated with lower attachment scores for children born preterm with either parent.

**Conclusion:**

The findings highlight the importance of including fathers in research and clinical practice and informing them about preterm birth, possible problems, and developmental consequences as well. Health professionals should be advised to create interventions focusing on both parents to enhance the quality of attachment in parent-child dyads in children born preterm.

## Introduction

Attachment, the emotional bond that develops between an infant and its caregivers, is shaped by interactions from an early age on. If attachment is not sufficiently established, children show a variety of problems [1]. Although current research has shown that multiple attachments begin to arise at a young age, and have an inherent potential to strengthen or compensate for attachment experiences with the primary caregiver, the majority of research concentrates on mother-child attachment for normally developing children while fathers, who make an important contribution to children’s life as well [2], and vulnerable children are rarely focused.

Children born preterm, i.e., born before the 37^th^ week of gestation, are considered as particularly vulnerable. Until now, research on mother-child attachment in children born preterm produced controversial results. Whereas some studies found no difference between children born preterm and children born at term regarding mother-child attachment [3, 4], others reported more disorganized attachment patterns in those born too early [5] or overall less secure attachment [6].

Potential risk factors for impairments in attachment can be found on both the parents’ and the child’s side including a higher risk for a variety of physiological issues, difficulties in emotional regulation [7] and attention deficits [8] in children born preterm compared to children born at term. Social behavior of children born preterm seems already atypical at an early age, indicated by shorter fixation times on social contents [9]. Furthermore, children born preterm show developmental problems in early childhood [10, 11], that may persist until adulthood [12]. Consequently, preterm birth has been associated with the vulnerable child syndrome [13], which usually refers to physical illnesses but may be observed for developmental issues as well. The term means that parents perceive a child as vulnerable although there is no reason even though there might have been one in the past. Persistent perception of vulnerability [14] as well as parental anxiety [15] after a preterm birth seem to be associated with less positive parent-child interaction and parental attitudes. Parents of children born preterm often fear possible handicaps [16] and therefore might focus on developmental delays which could affect the relationship.

On the parents' side, preterm birth increases the risk of anxiety, depression, and PTSD [17], and it requires more adaptive competence to compensate for children’s reduced responsivity [18], which could lead to insecure attachment. Furthermore, both mothers, as well as fathers of children born preterm, are more stressed than parents of terms [19]. The situation might be aggravated by the fact that a growing population among babies born preterm are twins as a result of infertility treatment. Meeting the needs of two vulnerable infants is even more challenging, which could lead to even higher rates of insecure attachment in twins [20].

Due to the factors described, impairments in interaction as the most frequent violation for secure attachment have been a focus in research about prematurity. Maternal sensitivity seems to be the same as for mothers of children born at term [21], but studies on the overall quality of interaction between children born preterm and their parents produced mixed results [22–24]. Several researchers have proposed that vulnerable children, in particular, profit from positive interaction and need even more positive input to reach the same levels as less vulnerable children [25]. This proposal might be transferable to the formation of mother-child as well as father-child attachment.

As described, the children themselves differ in many ways from healthy term children. Even though child age is adjusted with regards to prematurity when assessing child development, this does not seem to do justice to the differences completely. There seem to be long-lasting consequences [12, 26], indicating fundamental dissimilarities between children born preterm and at term, which have the potential to influence attachment. It seems as if it is not so much the parental behavior that is different, but more the child, that was born preterm, itself [24]. Therefore sensitivity, for example, does not seem to be as closely related to attachment in mother-child dyads with children born preterm as it is for those with children born after 37 weeks of gestation [21], suggesting that other variables should be considered when studying children born preterm.

Although there is only a small association between child development and mother-child attachment for infants born at term [27], this link seems to be stronger in children born preterm [5]. Most studies describe developmental issues as the base for potential problems in mother-child attachment in children born preterm, but they focus on different facets and do not examine (except [5]) the link in their research. Therefore this study includes child development and other characteristics as a base to describe attachment in children born preterm.

Paternal behavior seems to be more sensitive to environmental conditions than maternal behavior [28], and evidence suggests that child development and other characteristics might be especially relevant to fathers [29]. However, systematic research concerning this assumption is scarce. Fathers seem to demonstrate lower interaction quality with children born preterm than with children born at term and lower quality than mothers [23]. This is supported by relatively high levels of unresponsiveness in fathers of children born preterm compared to mothers [30]. Overall, father-child attachment has not been a particular focus in research about children born preterm. There seems to be no difference between fathers of children born before and at term when it comes to bonding after birth [31]. A study that examined attachment in a small sample of fathers of children born very low birthweight (<1500g) and at term found no differences in the distribution of attachment patterns [32].

The present study aimed to compare mothers and fathers of children born preterm and at term regarding parent-child attachment. This question is important for interventions in neonatal intensive-care units, targeting not only mothers but fathers equally. Furthermore, we tested if the effects of certain child characteristics on the parent-child relationship function differently between these groups. While controlling for socioeconomic status (SES), healthy children born preterm were compared with term children to answer two research questions: (1) Do preterm and term children differ in their attachment towards mothers and fathers? (2) Are child characteristics, such as twin, gestational, and developmental status as well as child gender predictive for children’s attachment?

## Methods

### Sample

The Ethics Committee of the Medical University of Vienna approved the research study (ECS 1710/2013), and parents provided written informed consent before participation. The sample consisted of 245 cohabiting fathers and mothers and their children, of whom 190 were born preterm (45 of them twin pairs) and 100 at term. In all families, the mother was the primary caregiver. All families with children that were born preterm and were between 12 and 36 months of age within the data collection period, born or treated at the clinic of the Medical University of Vienna/Austria, were contacted after a screening at the clinic. Exclusion criteria were cognitive or physical impairment, single- or step-parental care, or non-German speaking parents, which in total ruled out 43% of the children born in the hospital at the time. Out of the remaining families equal numbers in respect to gestational age, as well as gender of the child were drawn. About 20% of the families could not be reached due to missing or wrong contact information, and 22% of the contacted families refused to participate. Refusal was not connected to child age, gender, or gestational age at birth. To allow reliable estimation of the effect of being a twin, this group was oversampled within the target population. The reference group of children born at term (> 37 weeks of gestation) was recruited from playgroups and nursery schools in and around Vienna/Austria.

### Procedure

Data was collected between 08/2013 and 12/2015 as part of the CENOF Research Study [33]. All families were visited three times at home. Two times to observe the children once with their fathers and once with their mothers, in quasi-randomized sequence and a third time to assess child development.

### Measures

#### Demographic characteristics

Information on the family’s SES was obtained by structured interviews at the first visit to the family. Education, which was used as a control variable for all analyses, was grouped in academic and non-academic (high-school education or less) maternal or paternal education. Gestational age was reported by medical reports and entered the analysis as the severity of prematurity (prematurity), objectified by the number of days that the child was born before term. The assessment age was corrected for prematurity.

#### Assessment of attachment

The Attachment Q-Sort (AQS) [34] was used to describe the quality of the attachment relationship in a naturalistic setting. The AQS consists of 90 items describing children’s attachment behavior in the presence of another person. For example: “*Child readily shares with mother or lets her hold things if she asks to*.” or “*Child clearly shows a pattern of using mother as a base from which to explore*. *Moves out to play; returns or plays near her; moves out to play again*, *etc*.” Each item is rated on a 9 point scale. In the end, the specific ratings of a dyad are correlated with the ratings of an ideally securely attached child, providing a score between -1.0 to +1.0. Two trained observers observed the child the caregiver for two hours at home and independently rated the parent-child relationship. Inter-rater reliability was ICC = .91 for the maternal and ICC = .94 for the paternal AQS-score.

#### Assessment of development

Trained assessors completed the mental, language, and motor indices of the Bayley Scales of infant and toddler development, BSID-III [35], for all children during a home visit. The reliability coefficients over the different scales varied between .87 and .91 and the measure was reported as valid with regards to former versions of the Bayley Scales and other traditional tests measuring child development.

### Data analysis

#### Outlier detection and missing data handling

Outliers can severely compromise the integrity of analysis results [36]. Hence, a robust, multivariate outlier-detection algorithm [37] was applied. After grouping all cases into *n*/10 clusters, it regards all cases in clusters with *k* < 5 members as outliers because they are isolated within the multivariate feature space. Accordingly, the algorithm removed four children born at term and ten children born preterm. The remaining 276 cases had an overall missing rate of 3.7%, with a maximum of 10.1% on the maternal AQS-score for a single variable. In all further analyses, we dealt with missing data by relying on full information maximum likelihood (FML). A control analysis with all cases included changed the results only negligible.

#### Data modeling

Considering the mother-father-child triangle as an interdependent system both parent-child dyads were analyzed synchronously via path analysis to account for dependency. To avoid multicollinearity the BSID-III indices were bundled to a latent factor representing the overall child development status.

Interested in differences between mothers and fathers, but also between families with children born at term and before we subjected the model to a multi-group analysis allowing comparisons of all parent groups. However, the data structure across parent-child groups differed. In the preterm group, several families had twins and therefore contributed two cases to the analysis. To tackle the resulting within-variance, the base model was reformulated as a two-level model, but the analysis was kept limited to the between-level with the families as subjects. In the term group being a twin did not vary and prematurity varied only irrelevantly, so both effects were fixed to zero in this group. Due to those necessary restrictions chi-square based fit statistics were not computable for the full model. Statistical model comparison was still possible via Wald tests for parameters, and the *R*^2^ value of the parent-child attachments served as an overall fit measure.

To test for mean differences across parents, we restricted the model to function similar to traditional analysis of covariance with parent gender and birth status as factors and education of the parents and the child’s gender and age as covariates. Development and the extent of prematurity were not included due to their strong correlations with birth status. But, although those characteristics are tautological in a between-group comparison, they are informative for explaining the within group variance. To assess the differential impact of development and extent of prematurity on attachment in specific parent groups we extended the initial model to include those variables and allowed all co-variates to have varying weights across parents.

All main analyses were carried out with MPlus 7.1 [38] using robust maximum likelihood estimation (MLR) to deal with possible non-normality.

## Results

### Sample characteristics

After removing the 14 identified outliers, the overall sample consisted of 95 singletons and 85 twins born preterm and 96 mature singletons, with 139 boys and 137 girls (see Table 1) between 12 and 36 months (*M* = 19.4, *SD* = 6.0). All families were Austrian middle-class, with 38–61 percent of parents in the subgroups having an academic education.

**Table 1:** Demographics of participating children subjected to the main analysis.

	Total *n* = 276		Term Singletons *n* = 96		Preterm Singletons *n* = 95		Preterm Twins *n* = 85	
	*M* / %	95% CI	*M* / %	95% CI	*M* / %	95% CI	*M* / %	95% CI
Child gender female, %	49.6	43.7–55.5	53.1	43.1–63.1	50.5	40.5–60.6	44.7	34.1–55.3
Gestational age, weeks, *M*	33.6	32.9–34.2	39.9	39.7–40.2	29.4	28.6–30.2	31.0	30.4–31.7
Child age, months, *M*	19.1	18.4–19.8	19.3	18.2–20.4	18.6	17.4–19.9	19.4	18.1–20.7
Birthweight, gramms, *M*	2051	1922–2181	3359	3273–3445	1322	1186–1458	1436	1334–1539
Apgar Score (1 minute), *M*	7.9	7.7–8.1	9.0	8.9–9.1	7.3	6.9–7.7	7.6	7.3–7.9
Development, *M*								
Cognitive development	106.54	104.9–108.2	111.3	108.4–114.1	102.8	99.9–105.7	105.1	102.5–107.8
Language development	100.0	98.2–101.8	104.5	101.3–107.7	97.5	94.7–100.3	97.1	94.1–100.1
Motor development	101.2	99.5–102.9	106.9	103.9–109.9	98.0	95.3–100.7	97.5	94.9–100.2
Education, academic, %								
Mother	50.2	44.2–56.2	61.1	51.2–70.9	38.7	28.8–48.6	50.6	39.7–61.5
Father	49.6	43.7–55.6	56.8	46.9–66.9	45.7	35.7–55.8	45.8	35.1–56.5
Parent age, years, *M*								
Mother	34.1	33.5–34.7	33.6	32.6–34.6	34.5	33.4–35.6	34.1	33.0–35.3
Father	37.1	36.3–37.9	36.7	35.3–38.0	37.1	35.7–38.5	37.6	36.4–38.8

CI, 95% confidence interval.

### Mean differences of parent-child attachment

**Fig 1:**
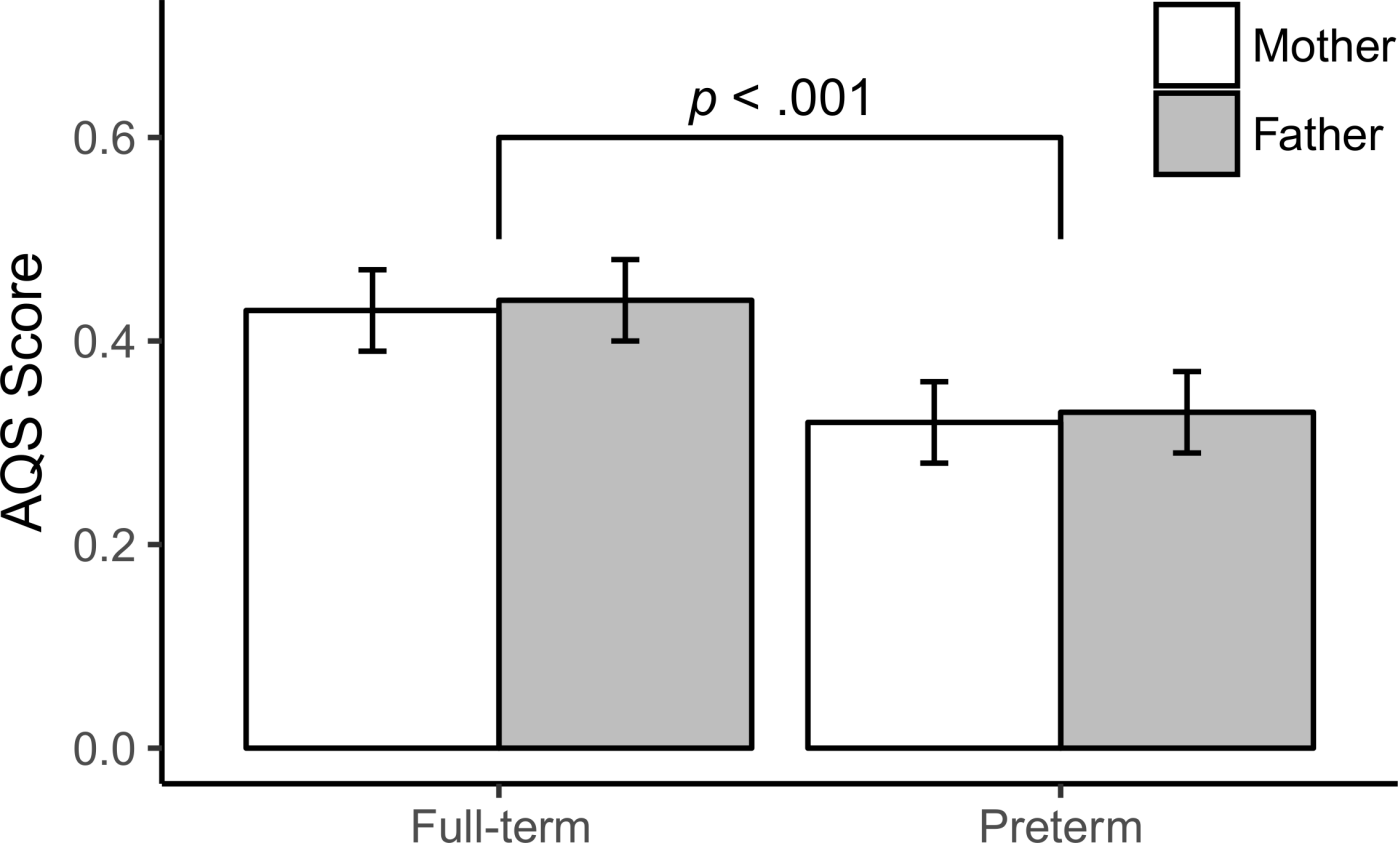
AQS-score group means of mothers and fathers of children born full-term or preterm.

In families with children born at term the estimated marginal mean of the AQS score was 0.43 (SE = 0.04) for mothers and 0.44 (SE = 0.04) for fathers. In families with children born preterm the mean for mothers was 0.32 (SE = 0.04) and for fathers 0.33 (SE = 0.04) (see Fig 1). Testing for the effects of parent gender and birth status on the AQS score no interaction effect of both factors was found, *Wald* = 0.02, *p* = .89, the main effect of birth status was statistically significant, *Wald* = 13.9, *p* < .001, *d* = 0.26, but fathers and mothers did not differ, *Wald* = 0.08, *p* = .78. Among the covariates, only the child’s gender was significantly associated with the AQS scores, *b* = 0.14, *p* = .003, favoring girls over boys.

### Child and parent characteristics linked to attachment

**Fig 2:**
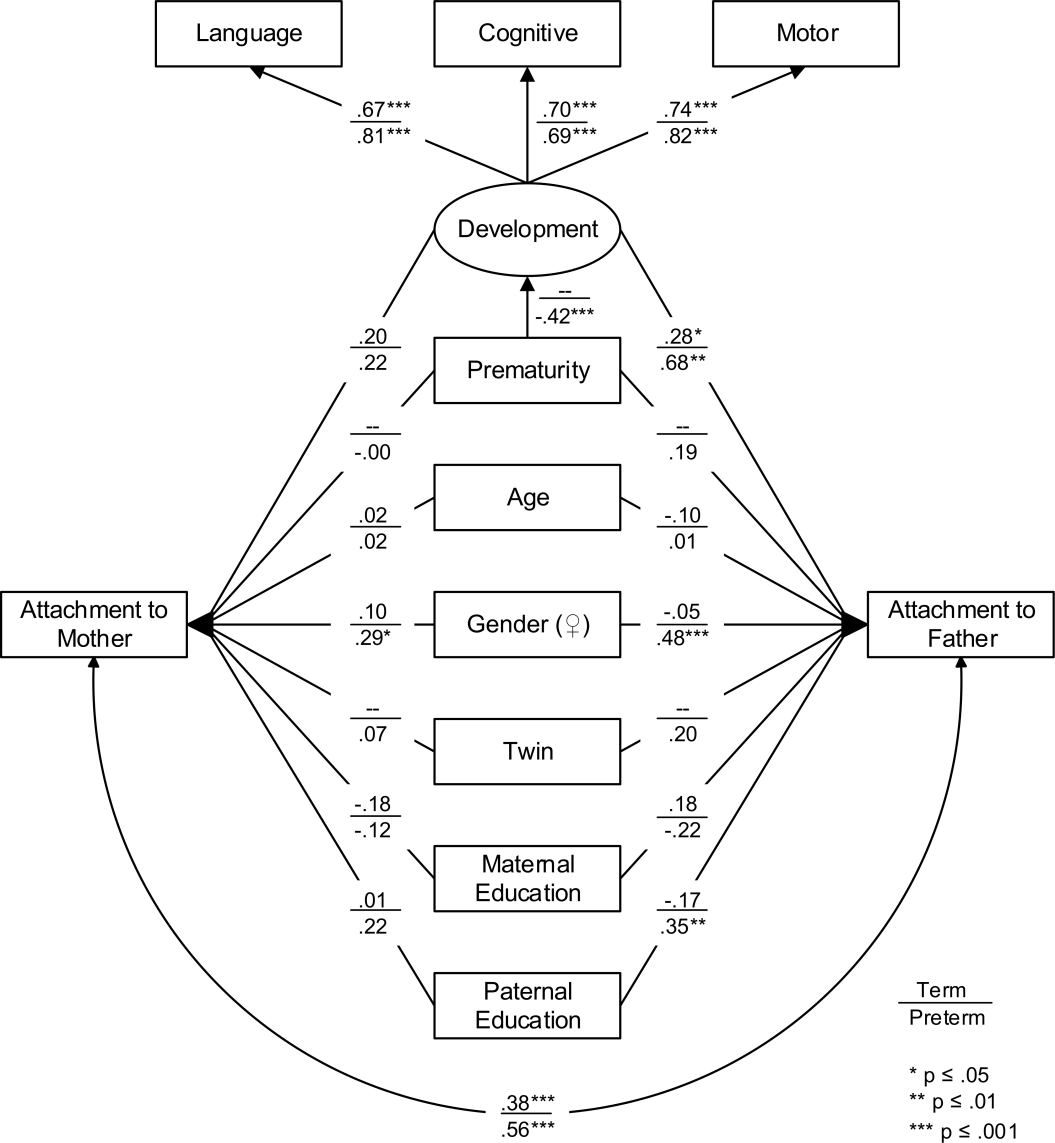
Multigroup base model of mother-child and father-child attachment in preterm and term children. Note: the weights of the BSID-III differ only due to standardization.

To test whether the predictors of the AQS score function differently between mothers and fathers as well as between children born term and preterm regression weights were compared between the four combinations. As a first step, a model with unconstrained regression path weights for all parent constellations was estimated. As shown in the overview of all path weights (see Table 2 and Fig 2), the nominally most substantial effects were found for fathers in the preterm group, while in mothers of children born preterm and in both parents in the term group the effects were much smaller and mostly statistically insignificant.

**Table 2:** Standardized path coefficients of unrestrained base model.

To	From	Term			Preterm		
		*β*	95% CI	*p*	*β*	95% CI	*p*
Development	Prematurity	0^a^			-.42	-.60–-.25	< .001
Maternal attachment	Development	.20	-.04–.44	.10	.22	-.08–.52	.16
	Prematurity	0^a^			-.00	-.24–.23	.98
	Twin	0^b^			.07	-.16–.31	.54
	Gender of child	.10	-.18–.37	.50	.29	.06–.52	.01
	Age of child	.02	-.17–.20	.87	.02	-.16–.20	.85
	Maternal education	-.18	-.39–.03	.09	-.12	-.39–.16	.42
	Paternal education	.01	-.21–.23	.95	.22	-.06–.49	.12
Paternal attachment	Development	.28	.06–.51	.01	.68	.24–1.00	.002
	Prematurity	0^a^			.19	-.09–.47	.18
	Twin	0^b^			.20	-.03–.43	.08
	Gender of child	-.05	-.32–.22	.70	.48	.21–.75	< .001
	Age of child	-.10	-.32–.11	.35	.01	-.24–.26	.97
	Maternal education	.18	-.01–.37	.07	-.22	-.48–.05	.11
	Paternal education	-.17	-.37–.03	.09	.35	.09–.61	.009

^a^ Fixed to zero because no meaningful effect is expected within the sample’s value range

^b^ Fixed to zero because predictor does not vary within sample

In the preterm group, three significant predictors for the paternal AQS score, namely child development, child’s gender, and academic education of the father, were found, whereas for the maternal AQS score only child’s gender displayed a significant effect (see Table 2). In the term group, the maternal AQS score was not significantly associated with any of the predictors, and the paternal AQS score only with development.

To statistically compare paths weights, we calculated Wald tests for all differences with at least Δ*β* ≥ |.2| between mothers and fathers within birth status groups and mothers and mothers as well as fathers and fathers across birth status groups. Comparing mothers and fathers, results indicated that the link between low development and low attachment scores was stronger for fathers than for mothers in the preterm group (*Wald* = 4.4, *p* = .036), but did not differ between fathers of children born preterm and at term (*Wald* = 1.7, *p* = .20). Between the preterm and term group, only the impact of the child’s gender (*Wald* = 4.0, *p* = .046) on the father-child attachment differed. Whereas children’s gender appeared to be irrelevant in the term group, father-son attachment scores were lower than father-daughter attachment in the preterm group. Being a twin did not affect attachment to either parent.

Overall, we found individually meaningful differences between groups only for a few paths. In sum, however, effects on attachment for fathers of children born preterm were consistently larger than for the three other parent-child constellations. This amounted to a massive difference in explained variance. The predictors for attachment to fathers of children born preterm accumulated to *R^2^* = .82, while for mothers only *R^2^* = .19, for fathers of children born at term *R^2^* = .15, and for mothers *R^2^* = .08 could be explained. Pooling those three together their *R*^2^ is distinctively smaller (*Wald* = 17.4, *p* < .001) than for fathers of children born preterm.

## Discussion

The present study investigated mother-child and father-child attachment in children born preterm and at term. While we found less secure attachment in children born preterm than in children born at term with both parents, the investigated predictors were most interesting for father-child attachment in the preterm group.

As described in the introduction, having a child born preterm poses a challenge for parents. From an evolutionary point of view investment in vulnerable children is less rewarding than investing in healthy strong offspring considering the trade-offs for parenting. That conflict is less prevalent in older parents like in the present sample with a small number of children, who live in a relatively secure environment [39, 40]. Despite these favorable conditions we still found differences between children born preterm and at term, regarding attachment security but also its predictors, which should be even more pronounced in samples with additional risks.

While there is little to no research on father-child attachment, the result of lower attachment scores in children born preterm with their parents stands in contrast to some previous findings [4] on mother-child attachment in smaller samples of children born preterm. Nevertheless, problems regarding attachment in mother-preterm dyads have been reported before [6].

With respect to the child factors, the only significant effect for mothers of children born preterm was seen for child’s gender, with higher scores for girls. In contrast to previous evidence [5], better development only showed a small but not significant association with mother-child attachment in the present study. This could be due to the use of the AQS, that captures attachment in the child’s daily life, instead of the Strange Situation Procedure [41], which measures attachment creating a stressful situation for the child. While the Strange Situation Procedure has many advantages, the AQS is a valid alternative and can be applied for a broader age range, can be easily repeated within a relatively short period and does not elicit stress [42], which might be especially relevant for children born preterm. Differences in attachment between children born preterm and at term have been discussed but not always found independent of which methods to measure attachment were used. Since to our knowledge, this is the first study using the AQS on a large sample of children born preterm, further research will be needed.

For mothers of children born at term, none of the child factors or parental education seemed to affect attachment quality. Research has so far mostly focused on this group, and some factors like sensitivity or reflective functioning are well established as bases for mother-child attachment [43, 44].

For fathers of children born at term, attachment was as well not related to child gender or parental education but positively to children’s development. This association was also found in preterm children, suggesting that for fathers the interaction with less developed children is more difficult than with well-developed children. Father-child communication is more challenging and associated with more breakdowns [45] than mother-child communication, which could be pronounced in less developed children. Interacting with children born immature, who are less responsive [18], might also lead to more struggling with interpreting cues on the father’s side. Therefore, better development could facilitate interaction and the formation of secure attachment.

It has been hypothesized that fathers stimulate and destabilize their children, whereas mothers care for and contain them [46]. This paternal behavior might seem less appropriate for less developed children, and the lack thereof could affect the relationship. Physical play, which is often mentioned within that context, occurs most often with boys and seems to be especially important for them [47]. This finding could be one explanation why a negative association between attachment of fathers and their male children born preterm was found. At least at birth, preterm boys are more vulnerable than girls [48], and problems in attachment at toddler-age might be still linked to that gender difference. Affect regulation and emotional communication are more problematic in father-son dyads compared to father-daughter dyads and mother-child dyads in general [49]. This could be a risk for preterm boys, who already show more problems after birth than girls do [50].

Being vulnerable is diametrically opposite to the male gender stereotype. Dependent and emotional behavior is discouraged in boys from an early age on [51], and people react negatively to males, who do not confirm gender stereotypes [52]. Men are believed to suppress emotions, which are inconsistent with their gender role [53] and are less likely to seek and receive support [51]. This poses a challenge for fathers with their own non-gender conform emotions after preterm birth and a child that might not fulfill male stereotypes. In general, fathers are the ones that differentiate more between boys and girls [54]. Investment in male preterms could be diminished since from an evolutionary point of view being strong and physically fit is more desirable in male than female offspring. The effect was therefore also found for mothers of children born preterm, suggesting that gender-expectations play a crucial role in mothers as well. Surprisingly, no significant effect of twin-birth was found on parent-child attachment, suggesting that the relationship is not more challenged by having two premature children compared to one.

For fathers of children born preterm all predictors together added up to 82% of explained variance in attachment, whereas in all other groups it did not exceed 20%. By detecting the relevant predictors of secure attachment for this group of fathers, that is, low paternal education, male gender of the child, and increased problems in development the identification of specific risk groups regarding attachment was achieved.

The strengths of this study were (1) the large sample size, including children of different gestational age and twins and singletons. Former studies of mother-child attachment in children born before term have not integrated all these factors. Furthermore, (2) the AQS was employed instead of the traditional Strange Situation Procedure, which has been questioned in its use for children born preterm since they are particularly vulnerable to stress [5, 55]. (3) We also investigated mother-child and father-child attachment, while most previous studies focused exclusively on mothers.

The study presented here has several limitations. The parents within the sample were from middle to high SES and volunteered to participate in this study, which required considerable cooperation. The sample was selected retrospectively, and we only included families, where both parents were living together with their healthy biological child. The present study is, therefore, just based on a specific subsample of families and we do not know whether the results can be generalized to families with lower SES. Because gender-typed behavior seems more common in less educated families [56], differences between mothers and fathers but also effects of the child’s gender might be pronounced in samples with less educated participants.

A further limitation might be the oversampling of twins within the target population. However, we carefully accounted for the effect of being a twin in the model and found it to be non-significant. Hence, we believe this has no negative impact on the generalizability. Overall, we cannot be certain whether the observed effects are persistent over time, or whether attachment within the preterm group becomes more secure with increasing child age.

## Conclusion

The present results emphasize the importance of paying special attention to parents of children born preterm and especially highlight the vulnerability of father-child attachment to child factors. We could show that children born preterm display problems regarding attachment with both parents. The children in this study were between 12 and 36 months old, which covers a wide range and shows that problems seem to persist. Interestingly in particular father-child attachment was related to basic child characteristics. With regard to attachment, the combination of fathers without an academic education and sons with lower development scores were identified as a risk group that will need increased attention in the future. Health professionals working with families of children born preterm are advised to create specific conditions for fathers and provide ongoing support to accommodate their particular needs. It will be important to develop interventions focusing on gender-specific developmental pathways in children born preterm in order to enhance especially the quality of attachment in father-son dyads. Gender-typed expectations or ideas about children’s development and behavior should be addressed as part of the work with both parents, but in particular with fathers.

## Supporting information

S1 Data File (SAV)
